# Erythrocyte Membrane-Coated Arsenic Trioxide-Loaded Sodium Alginate Nanoparticles for Tumor Therapy

**DOI:** 10.3390/pharmaceutics12010021

**Published:** 2019-12-24

**Authors:** Yumei Lian, Xuerui Wang, Pengcheng Guo, Yichen Li, Faisal Raza, Jing Su, Mingfeng Qiu

**Affiliations:** School of Pharmacy, Shanghai Jiao Tong University, Shanghai 200240, China; lym-0517@sjtu.edu.cn (Y.L.); wangxuerui0303@163.com (X.W.); gpcedu@sjtu.edu.cn (P.G.); liyichen592@sjtu.edu.cn (Y.L.); faisalraza@sjtu.edu.cn (F.R.)

**Keywords:** red blood cells membrane, arsenic trioxide, sodium alginate nanoparticles, reduce toxicity, anti-tumor

## Abstract

Arsenic trioxide (ATO) has a significant effect on the treatment of acute promyelocytic leukemia (APL) and advanced primary liver cancer, but it still faces severe side effects. Considering these problems, red blood cell membrane-camouflaged ATO-loaded sodium alginate nanoparticles (RBCM-SA-ATO-NPs, RSANs) were developed to relieve the toxicity of ATO while maintaining its efficacy. ATO-loaded sodium alginate nanoparticles (SA-ATO-NPs, SANs) were prepared by the ion crosslinking method, and then RBCM was extruded onto the surface to obtain RSANs. The average particle size of RSANs was found to be 163.2 nm with a complete shell-core bilayer structure, and the average encapsulation efficiency was 14.31%. Compared with SANs, RAW 264.7 macrophages reduced the phagocytosis of RSANs by 51%, and the in vitro cumulative release rate of RSANs was 95% at 84 h, which revealed a prominent sustained release. Furthermore, it demonstrated that RSANs had lower cytotoxicity as compared to normal 293 cells and exhibited anti-tumor effects on both NB4 cells and 7721 cells. In vivo studies further showed that ATO could cause mild lesions of main organs while RSANs could reduce the toxicity and improve the anti-tumor effects. In brief, the developed RSANs system provides a promising alternative for ATO treatment safely and effectively.

## 1. Introduction

Arsenic trioxide (ATO) is the main active ingredient of traditional Chinese medicine (TCM) Arsenic. In the 1970s, it was first applied to acute promyelocytic leukemia (APL) with significant efficacy [1] and was approved by the National Medical Products Administration (NMPA) and Food and Drug Administration (FDA) as a first-line treatment for APL in 1999 and 2000, respectively [2,3]. ATO can induce cell differentiation, inhibit apoptosis, and exert anti-tumor effect [4]. In recent years, research studies have confirmed the significant growth inhibition and apoptosis induction effect of ATO in solid tumors, such as liver cancer, breast cancer, stomach cancer, glioma and lung cancer [5,6,7,8,9,10,11]. At present, ATO injection has been employed clinically in the treatment of APL and advanced primary liver cancer. However, the unique physicochemical properties of ATO allow it to be rapidly cleared from blood, and it requires daily administration during clinical treatment. At the same time, the uptake of the reticuloendothelial system (RES) makes only a slight amount of ATO reach the tumor site. Nevertheless, considering the potent toxicity of ATO, increasing the dose of ATO will increase the systemic toxicity and cause damage to the liver, kidney, heart, and peripheral nerve [12,13,14].

Based on the size advantage, nanoparticles (NPs) can exude through the tumor vasculature and effectively delivery the drugs to cells through enhanced permeability and retention (EPR) effects [15]. Therefore, it is considered to be a kind of formulation with low toxicity and high stability. Different ATO delivery systems (DDS) have been developed, including magnetic nanoparticles [16], chitosan nanoparticles [17], microspheres [18], liposomes [19], and mesoporous silica nanoparticles [10,20]. These formulations can achieve sustained release of ATO, which can reduce the transient plasma concentration and toxicity of drugs to a certain extent. However, they are still deficient in biocompatibility, and the safety of these systems needs to be verified [21].

Sodium alginate (SA) is a sort of polyanionic polysaccharide alginic acid salt found in brown algae that is water-soluble and has the advantages of anti-tumor effect, immune regulation, non-toxic, biodegradability, and excellent biocompatibility [22,23]. It has been approved by FDA for the pharmaceutical industry as an excipient [24,25]. For the past few years, NPs prepared from SA as drug carrier systems have also attracted more attention [26,27]. Red blood cells membrane (RBCM) will be formed into vesicles (RVs) using extrusion or sonication methods [28]. As a drug carrier, it can be attached to the surface of NPs to sustain the release of drugs, avoid elimination by the immune system, increase drug stability, improve biocompatibility and thus prolong drug circulation in vivo [29,30].

Moreover, RBCM coating nanotechnology already has excellent precedents. Che-Ming J et.al [31] demonstrated the synthesis of an RBCM coated polymeric nanoparticle for long-circulating cargo delivery, Jinghan Su et al. [32,33,34] extensively studied the effectiveness of RBCM-camouflaged NPs for treating metastatic breast cancer. In addition, if the non-toxic SA nanoparticles can be encapsulated by natural RBCM and combine the superiorities of sustained release and prolonging residence time, the nano-system can achieve the purpose of maintaining efficacy and reducing toxicity. In addition, this can provide a new possibility for safe application of ATO.

**Figure 1:**
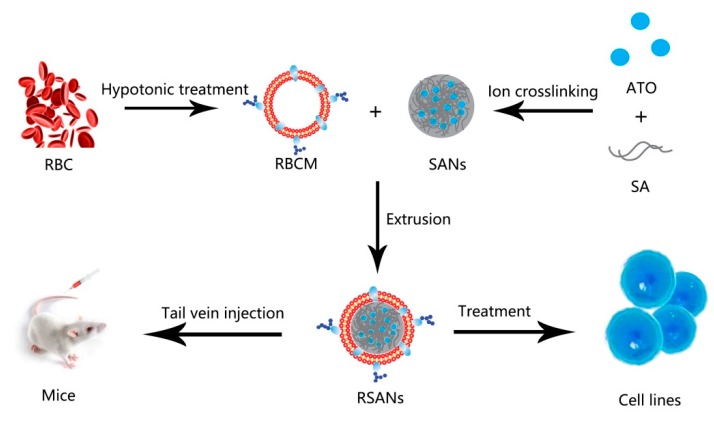
Preparation and Characterization of RSANs.

In this study, RBCM-camouflaged ATO-loaded sodium alginate nanoparticles (RBCM-SA-ATO-NPs, RSANs) were prepared as shown in Figure 1. ATO-loaded sodium alginate nanoparticles (SA-ATO-NPs, SANs) were prepared by the ion crosslinking method, followed by coating of RBCM to obtain RSANs. It was then systematically characterized and evaluated for its efficacy and toxicity. The results indicated that the system might become a promising delivery system for the safe, effective, and sustained release of ATO.

## 2. Materials and Methods

### 2.1. Materials

SA (200–500 Pa·s) was purchased from Shanghai Taitan Technology Co., Ltd., Shanghai, China, anhydrous calcium chloride (CaCl_2_) was purchased from Shanghai Lingfeng Chemical Reagent Co., Ltd., Shanghai, China and Polosham 188 (F-188) was purchased from Shaanxi Zhengyi Pharmaceutical Accessories Co., Ltd. Carbon support copper mesh (230 mesh) and phosphotungstic acid were obtained from Beijing Zhongjing Keyi Technology Co., Ltd., Beijing, China. 1,1′-dioctadecyl-3,3,3′,3′-tetramethylindocarbocyanine perchlorate (Dil, cell membrane green fluorescent probe), Hoechst 33342, 4% paraformaldehyde fix solution, antifade mounting medium and Cell Counting Kit-8 (CCK-8), were all purchased from Biyuntian Biotechnology Co., Ltd., (sShanghai, China) 5(6)-aminofluorescein was bought from Nanjing Xinfan Biotechnology Co., Ltd., Nanjing, China. Polycarbonate film was bought from Whatman Company, City, UK. Dialysis bag (Cut-off molecular weight = 3500Da) was obtained from United States for carbonization. Fetal bovine serum (FBS), RPMI 1640 medium, and DMEM medium were ordered from the Shanghai Chenyi Biotechnology Company, Shanghai, China. Trypsin and penicillin-streptomycin were purchased Yingjie Jieji (Shanghai) Trading Co., Ltd., Shanghai, China.

### 2.2. Cells and Animals

RAW264.7 cells (mice macrophages) and HEK-293 cells (normal human embryonic kidney cells) were bought from the Shanghai Cell Bank of the Chinese Academy of Sciences, Shanghai, China, and cultured with DMEM complete medium. SMMC-7721 cells (human liver cancer cells) and NB4 cells (APL cells) were obtained from Shanghai Jihe Biotechnology Co., Ltd., Shanghai, China and cultured with RPMI 1640 complete medium. Culture of the cells was performed in an incubator kept at 5% CO_2_ and 37 °C. The male BALB/c nude mice (SCXK 2017-0005) were obtained from Shanghai Slack Laboratory Animals Co., Ltd., Shanghai, China and kept in the Specific Pathogen Free (SPF) animal room of School of Pharmacy, Shanghai Jiao Tong University. Guidelines for care and use of laboratory animals of Shanghai Jiao Tong University were used to perform animal studies and these studies were duly approved by the animal ethics committee of Shanghai Jiao Tong University (No: A2019046, Date: 5 July 2019).

### 2.3. Preparation of SANs

SANs were prepared by the ion crosslinking method. In brief, 1.5 mL of 2 mg/mL CaCl_2_ was slowly added into 10 mL of 0.3 mg/mL SA (pH = 5) under stirring. After sonication for 5 min at 250 W, 0.1 mL of 10 mg/mL F-188 was added and then stirred for 30 min. To obtain SANs, 0.2 mL of 8 mg/mL ATO was added and stirred for other 30 min.

### 2.4. Preparation of RBCM

RBCM was extracted by hypotonic rupture method. In brief, whole blood of SD rats (bought from Shanghai Jiesijie Experimental Animal Co., Ltd., Shanghai, China) was collected through abdominal aorta. The blood was centrifuged (2000 rpm, 5 min, 4 °C), to obtain red blood cells (RBCs), and then washed with 1× phosphate buffer saline (1× PBS) for 3 times. To collect RBCM, 900 μL EDTA (0.2 mM) was added to disrupt the RBCs, followed by centrifugation (13,200 rpm, 10 min, 4 °C), and the above steps were repeated until the supernatant turned colorless. The obtained RBCM was resuspended in EDTA, and then stored in −80 °C refrigerator.

### 2.5. Preparation of RSANs

The prepared RBCM was sonicated at 250 W for 3 min to obtain RVs, and then sequentially extruded through polycarbonate films of 800 nm, 400 nm, and 200 nm by LF-50 extruder (Avestin Inc, agented by Shanghai Narujie Biotechnology Co., LTD, Shanghai, China) for at least 15 times respectively. The solutions of RVs and SANs were mixed at a ratio of 1:8(*v*/*v*). The prepared mixture was then extruded through polycarbonate films of 400 nm and 200 nm at least ten times, respectively, to obtain RSANs.

### 2.6. Characterization and Stability Test

The particle size and polydispersity index (PDI) of SANs and RSANs were determined by Malvern Zetasizer (He-Ne, 4.0 Mw, λo = 633 nm, Marvin instruments Ltd., Marvin, United Kingdom. The stability test was investigated simultaneously. The particle size and PDI of SANs and RSANs were measured for 15 days consecutively at both 4 °C and 37 °C.

### 2.7. Morphological Observation

Transmission electron microscopy (TEM, Thermo Fisher Scientific, Shanghai, China) was performed to evaluate the morphology of RSANs. 10 μL sample solutions were dropped on carbon-supported copper, air-dried and then rinses with 10 μL ultrapure water. Drip 10 μL 2% phosphotungstic acid to stain the samples, and the excess dye was removed with filter paper from the edge. After baking 30 min under an infrared baking lamp, the morphology was observed by TEM.

### 2.8. Drug Loading Capacity and In Vitro Release Study

The release study of ATO encapsulated in nanoparticles was performed in 1×PBS (pH 7.4). 2 mL ATO solution (final concentration of 135.6 μg/ mL), SANs, and RSANs solution were placed into dialysis bags, respectively (M_w_ = 3500). The dialysis bags were fastened at both ends and maintained under sink conditions at 37 °C using 50 mL PBS, then magnetically stirred at 100 rpm. At time point of 3 h, encapsulation efficiency (EE) and drug loading capacity (DL) of the nanoparticles were analyzed by extracting 1 mL release solution. The optimal DL and EE were investigated by adding different concentrations of ATO. For the determination of in vitro release, 1 mL release sample was taken at 0.5, 1, 2, 4, 8, 12, 24, 36, 48, 72, and 84 h, and then replaced with 1 mL of fresh PBS. The aliquots were filtered through 0.22 μm microfiltration membrane and diluted up to 50 times. Inductively coupled plasma-atomic emission spectroscopy (ICP-AES) was used to detect the concentration of ATO. In addition, the following formulas were used to calculated DL and EE.

Encapsulation Efficiency (%) = (*M*_1_ − *M*_2_)/*M*_1_ × 100%

Drug Loading Capacity (%) = (*M*_1_ − *M*_2_)/(*M*_1_ − *M*_2_ + *M*_3_) × 100%

where *M*_1_ means the total amount of used ATO, *M*_2_ is the amount of ATO in the dialysis solution, and *M*_3_ is the amount of used SA.

### 2.9. Hemolysis Test

Since it is administered by intravenous injection (iv), it is necessary to ensure that the nano-preparation will not cause hemolysis or cell aggregation. RBCs from the blood of SD rats were collected and normal saline was added to obtain a 2% (*v*/*v*) RBC suspension. The water, normal saline, RBC suspension, and RSANs were mixed into 12 tubes according to the ratio shown in Table 1. Tube 1 (RSANs) replaced with ultrapure water served as a positive control, while tube 2 (RSANs) replaced with saline as negative control. The results after incubation of 3 h and 24 h at 37 °C were recorded. Meanwhile, the absorbances of supernatant were determined at 3 h and 24 h through an ultraviolet spectrophotometer at the wavelength of 540 nm, and then the percentage of hemolysis was calculated according to formula:

Percentage of Hemolysis (%) = (*A*_sample_ − *A*_tube 2_)/(*A*_tube 1_ − *A*_tube 2_)

### 2.10. Macrophage Uptake Study

The immune escape ability of nanoparticles was investigated through RAW 264.7 cells. SA was labeled with 5(6)-aminofluorescein, and SANs and RSANs were prepared with labeled SA. A 12-well plate (2 × 10^5^ cells per well) was used for 24 h incubation. Serum-free medium was used to starve cells for 1 h and then replaced by complete medium containing SANs or RSANs (SA concentration was 50 μg/mL). A complete medium without nanoparticles was used as blank group. The cells were observed with laser scanning confocal microscope (LSCM) after incubation for 2 h. Before being photographed, the cells were fixed with 4% paraformaldehyde. The excitation wavelength of 5(6)-aminofluorescein was 493 nm. Also, three parallel groups were set up to detect the uptake quantitatively. After incubation with the drugs, each group was first digested with trypsin and then washed with PBS for 3 times, then resuspended in 500 μL PBS. The detection was performed through the FITC channel of a flow cytometer.

### 2.11. In Vitro Cellular Uptake

To investigate the uptake of nano-formulations by tumor cells and the structural integrity of NPs during this process, the test was implemented on NB4 and 7721 cells. The determination method was described in Section 2.10. For the qualitative detection, RSANs were prepared with labeled SANs and RVs (the RVs was labeled with Dil). The density of 7721 cells was 2 × 10^5^ cells per well, and the cells were cultured for 24 h. The cells were then treated in the same way as Section 2.10. After an incubation of 2 h, Hoechst 33342 was used to stain the nuclei for 15 min, and then the uptakes were observed with LSCM. The cells were fixed with 4% paraformaldehyde before being photographed. Since NB4 cells is a kind of suspension cells, the density of inoculation was doubled to avoid loss during the experiment, and finally immobilized on a glass slide coated with polylysine. The other steps were the same as the 7721 cells. The excitation wavelengths of 5(6)-aminofluorescein, Dil and Hoechst 33342 were 493 nm, 549 nm, and 405 nm, respectively.

### 2.12. Cytotoxicity Test

To investigate the toxicity of blank carrier material, the prepared SA nanoparticles (SNs) and RBCM-SA nanoparticles (RSNs) without ATO were diluted with DMEM complete medium to obtain SA concentrations of 8, 12, 20, 30, 40, 50, 60 μg/mL respectively. To investigate the toxicity of the nanoparticles after drug loading, free ATO solution, SANs, and RSANs were diluted to obtain different concentrations of ATO (1, 2, 4, 6, 8, 10, 12, 20 μg/mL). The above groups were administration groups (AG). 100 μL 293 cells (5 × 10^4^ cells/mL) were placed into a 96-well culture plate for 24 h. The nutrient medium was then replaced with 100 μL AG. At 24 h time interval, all the groups were cultured with 100 μL CCK-8 for 2 h. Only DMEM medium was set as a blank group (BG) and only 293 cells were set as a control group (CG). The optical density (OD) of samples were determined at 450 nm by a microplate reader and the following formula was used to calculate the cell viability.

Cell viability (%) = (ODAG − ODBG)/(ODCG − ODBG) × 100%

### 2.13. In Vitro Efficacy Study

NB4 and 7721 cells were selected to evaluate efficacy. 100 μL NB4 and 7721 cells (5 × 10^4^ cells/mL) were inoculated in a 96-well culture plate overnight, respectively. Then free ATO solution, SANs, and RSANs were administered to the cells, respectively. The concentrations of ATO were diluted to 1, 2, 4, 6, 8, 10, 12, 20 μg/mL. After 24 h, 100 μL CCK-8 were administered to evaluate the cell viability. To further investigate the inhibitory effect, the concentration of the ATO of each group was fixed at 1 μg/mL. The OD was measured after incubation of 4, 8, 12, 24, 36, 48, and 60 h, and the cell viability was determined. The blank and control groups were same as mentioned in Section 2.12.

### 2.14. In Vivo Toxicity and Safety Test

Due to the potent toxicity of ATO, the administration concentration at a safe level should be determined at first. For 2 weeks, ATO with high (40 μg/mL), medium (20 μg/mL) and low concentration (10 μg/mL) was administered through the tail vein respectively once a week at a dose volume of 0.2 mL per mouse. The mental state and death of nude mice were registered during the period.

The aim of the safety trial was to investigate whether the continuous intravenous injection of RSANs would cause lesions on systemic, hematological, and major organs. Afterward, the healthy nude mice were divided into the saline group, ATO group, SANs group, and RSANs group. The drugs were administered once every 2 days at dose volume of 0.2 mL per mouse and repeated seven times. The weight of the mice was recorded at 1, 3, 5, 7, 9, 11, 13 days after administration. Meanwhile, the mental state and death of mice were observed during the process. 2 days after the last administration, orbital blood was collected into the tubes, pre-mixed with heparin sodium, and white blood cells (WBC), glutamate pyruvic transaminase (ALT), aspartate aminotransferase (AST) were analyzed. In addition, after the mice were sacrificed by CO_2_ asphyxiation, the principal organs were excised. The viscera coefficients were calculated after weighing. Furthermore, the tissues were fixed with paraffin solution for immunohistochemical analysis (H & E) to examine its structure and morphology.

Visceral coefficient = weight of organ/body weight

### 2.15. In Vivo Anti-Tumor Studies

Male nude mice 6–8 weeks old with 7721 cells were used to investigate the anti-tumor effects of our nanoparticles. First we established xenograft tumor model. For the establishment of tumor model, 7721 cell suspension (2 × 10^7^ cells / mL) in a volume of 0.2 mL was injected subcutaneously in the armpit of the upper limb. In addition, then the formula width^2^ × length/2 was used to calculate the tumor volume every other day. At tumor volume of 100–250 mm^3^, then mice were grouped into four treatment groups (*n* = 5): (1) saline, (2) ATO, (3) SANs, and (4) RSANs. The drugs were administered intravenously at a dose of 1.3 μg/g every two days and repeated seven times. The CG was given normal saline for seven times. At the same time, the body weight and the tumor volume were recorded to evaluate for tumor inhibition efficacy as well as systemic toxicity. Two days after the last administration, the mice were sacrificed by CO_2_ asphyxiation, and the tumors were excised from each animal. The tumors were rinsed with normal saline, dried, and photographed, and the average tumor weight of each group was determined.

### 2.16. Statistics and Data Analysis

Data expression was shown as ± SD of the mean. Significant differences between SANs and RSANs were analyzed by Tukey Kramer multiple comparison tests, using GraphPad Prism Software, v.6.01 (GraphPad Software, Inc.). Results with *p* < 0.05 were considered significant and very significant with *p* < 0.01.

## 3. Results and Discussion

### 3.1. Characterization and Stability Test

**Figure 2:**
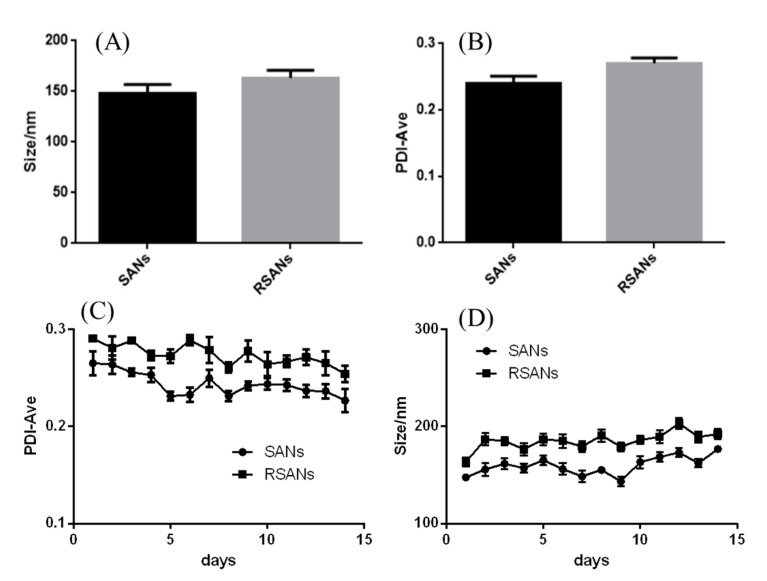
Particle size (**A**) and PDI (**B**) of SANs and RSANs. Changes of particle size (**D**) and PDI (**C**) within 15 days. Data are shown as ± SD (*n* = 3) of mean (*n* = 3).

The average size of RSANs was found to be 163.2 ± 4.4 nm (Figure 2A), which increased by about 15 nm as compared to SANs (147.9 ± 5.1 nm). The increment was consistent with the thickness of RBCM [35]. The PDI of SANs and RSANs was 0.24 and 0.27, respectively (Figure 2B), which indicated that NPs were well dispersed. In addition, the particle size of the nanoparticles was stable under 200 nm despite a slight increase in 15 days (Figure 2C) under the storage condition of 37 °C. The PDI remained below 0.3 (Figure 2D). These results revealed that NPs were stable. The structure and distribution were also uniform after 15 days. However, while the nanoparticles were stored under condition of 4 °C, the particle size increased significantly within 4 days and the flocculation occurred because of the obvious drug precipitation. It suggested that 37 °C may be a better storage condition. Moreover, after incubating the nanoparticles with serum for three days, no significant precipitation was observed, indicating that the nanoparticles can remain stable under serum conditions as well.

### 3.2. Morphological Observation

**Figure 3:**
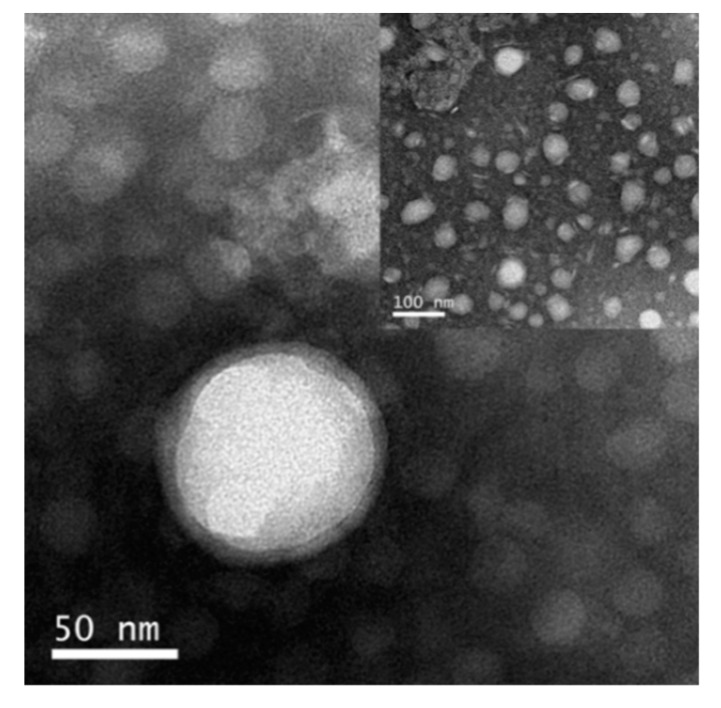
The TEM images of RSANs.

TEM showed that RSANs (Figure 3) were mostly spherical and uniformly distributed. RSANs presented a complete core-shell structure, which demonstrated that the RBCM successfully wrapped SANs. The particle size measured by TEM was about 50–100 nm, and it was smaller than the results obtained by Zetasizer. The difference might be due to the elimination of the water film outside the nanoparticles after the samples were dried.

### 3.3. In Vitro Release Study

**Figure 4:**
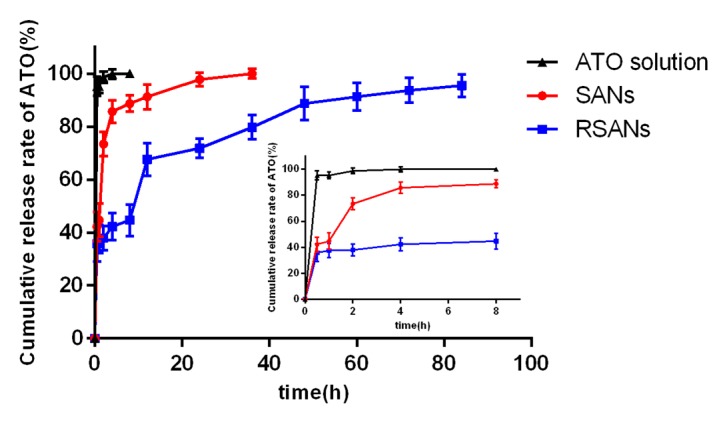
In vitro release curve of ATO, SANs and RSANs at 37 °C for 84 h. Data are shown as ± SD of the mean (*n* = 3).

Analyzed by ICP-AES, the average EE and DL were 14.31% and 4.98%, respectively (Table S1). As shown in Figure 4, the release of 3 groups was fast for 4 h. For the ATO group, the highest burst release of 98.61% was observed within 2 h, and all ATO was released in 4 h. After 4 h, the release of the SANs was slightly slow down with a cumulative release rate of 91% in 12 h, and the drug was completely released at 36 h. The release of RSANs was further slowed down to 67% at 12 h. The drug was then gradually released until the cumulative release rate of 95% was achieved at 84 h. Resultantly, RSANs showed more significant sustained release than SANs. The result revealed that forming a physical barrier around the nanoparticles by RBCM can significantly control the drug release.

### 3.4. Hemolysis Test

**Figure 5:**
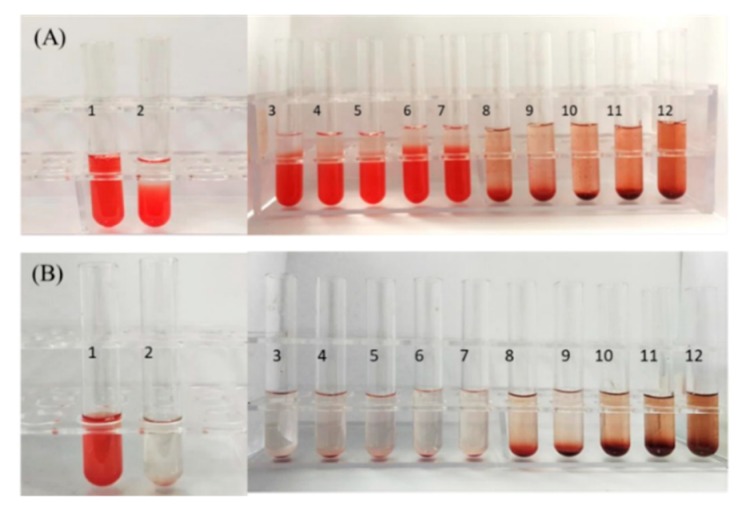
In vitro hemolysis results of RSANs after incubation of 3 h (**A**) and 24 h (**B**). Tube 1 was ultrapure water group, and tube 2 was the physiological saline group.

As RSANs are administered intravenously, the nanoparticles must not cause the rupture of RBCs. As shown in Figure 5A,B, the positive CG (Tube 1) was completely red and transparent with no cell. This confirmed that the RBCs were ruptured and causes hemolysis. For RSANs group, with the increase in concentration, RBCs sank, and the supernatants become colorless and clear from tube 1 to tube 7 similar to the negative CG (Tube 2). Further observation showed that no erythrocyte was aggregated under the inverted microscope. However, when the concentration increased gradually, slight hemolysis and hemagglutination phenomena were observed (tube 8 to tube 12). Results of hemolysis rate measurement were consistent with visual observation. The percentage of hemolysis was below 0.5% at high concentration, while it was below 0.01% at low concentration. The hemolysis test implied that RSANs were safe and suitable for the intravenous injection at final concentration of ATO lower than 0.0375 mg/mL.

### 3.5. Macrophage Uptake Study

**Figure 6:**
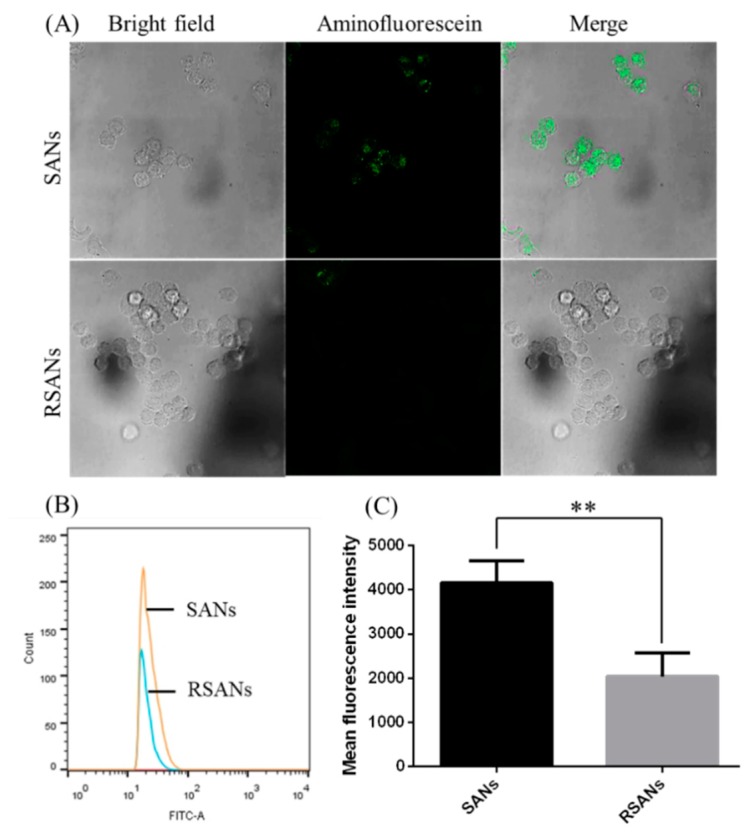
Cellular uptake of SANs and RSANs on RAW 264.7 macrophages. (**A**) Confocal image 630×. The SANs were labeled with 5(6)-Aminofluorescein (green), (**B**) Fluorescence intensity detection by flow cytometry. (**C**) Quantitative analysis of the fluorescence intensity. Data are shown as ± SD of the mean (*n* = 3). ** correspond to *p* < 0.01.

After the preparation of RSANs, it was necessary to confirm whether the biological activity of RBCM retains. The character of immune escape is one of the essential biological characteristics. The ability of RSANs for immune evasion was verified using confocal microscopy and flow cytometry by investigating the uptake between SANs and RSANs on RAW 264.7 cells. As shown in Figure 6A, the fluorescence intensity of RSANs groups observed by confocal microscopy was significantly weaker than that of SANs group. Meanwhile, the average fluorescence intensity of SANs and RSANs determined by flow cytometry were 4158.5 and 2045 respectively (*p* < 0.01), which was consistent with confocal results. The study confirmed that encapsulation by RBCM could help the nanoparticles to avoid the uptake of macrophages that can be related to special proteins such as CD47 [36,37]. It can be speculated that RSANs can also be prevented from being ingested by macrophages in vivo, and thus avoid the premature elimination of the drug.

### 3.6. In Vitro Cellular Uptake

**Figure 7:**
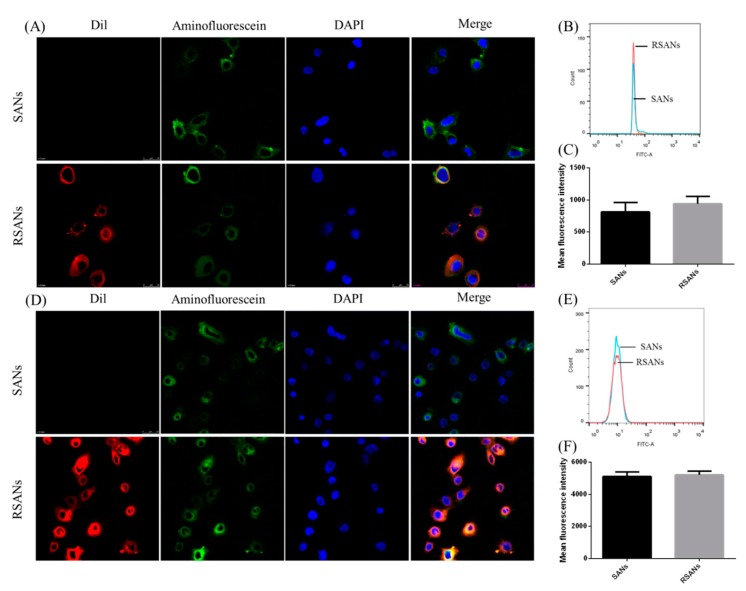
Cellular uptake of SANs and RSANs on NB4 cells and 7721 cells. (**A**,**D**) Confocal images 630×. The nucleus of cells was labeled by Hoechst 33342 (blue), SANs were labeled by 5(6)-Aminofluorescein (green) and RBCM were labeled by Dil (red). (**B**,**E**) Fluorescence intensity detection by flow cytometry. (**C**,**F**) Quantitative analysis of the fluorescence intensity. Data are shown as ± SD of the mean (*n* = 3).

To further confirm the in vitro cellular uptake and structure of RSANS, RBCM, SANs core and cell nucleus of NB4 cells as well as 7721 cells were labeled with Dil, 5(6)-Aminofluorescein and Hoechst 33342, respectively. As shown in Figure 7A,D, the red, green, and blue fluorescence represent the stained RBCM, SANs, and nuclei, respectively. After incubation of NB4 cells and 7721 cells with SANs and RSANs, it was detected that both red and green fluorescence overlapped around the nuclei. This indicated that both SANs and RSANs could be taken up by NB4 cells as well as 7721 cells. In addition, it could be preliminarily speculated the integrity of the basic shell-core structure was also maintained during the process. According to flow cytometry results, the average fluorescence intensity of SANs and RSANs ingested by NB4 cells were 813 and 941 (Figure 7B,C), while those of 7721 cells were 5111 and 5211 (Figure 7E,F) respectively. Both confocal and flow cytometry results showed that the uptake of RSANs was slightly increased without any significant difference. It can be predicted that the change of the negative charge on the surface of the nanoparticles after being coated by RBCM may cause the slight difference, which changed the repulsive force between membranes.

### 3.7. ATO Nanoparticles Cytotoxicity

**Figure 8:**
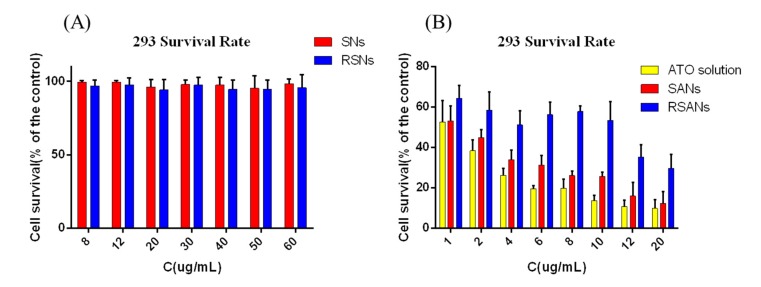
Cell survival of 293 cells after administration with different concentrations of (**A**) SNs and RSNs, (**B**) ATO, SANs and RSANs for 24 h. Data are shown as ± SD of the mean (*n* = 3).

In vitro cytotoxicity assays were performed to evaluate the safety of nanoparticles initially. As shown in Figure 8A, the cell viability of SNs and RSNs were both higher than 95% at SA concentration of 8–60 μg/mL. It indicated that SA and RBCM were not significantly toxic and had excellent biocompatibility. Free ATO revealed potent cytotoxicity at different concentration; however, the toxicity was reduced significantly after being encapsulated in nanoparticles (Figure 8B). It can be predicted that the nano-delivery system newly designed can remarkably improve drug safety during medical treatment.

### 3.8. In Vitro Efficacy Study

**Figure 9:**
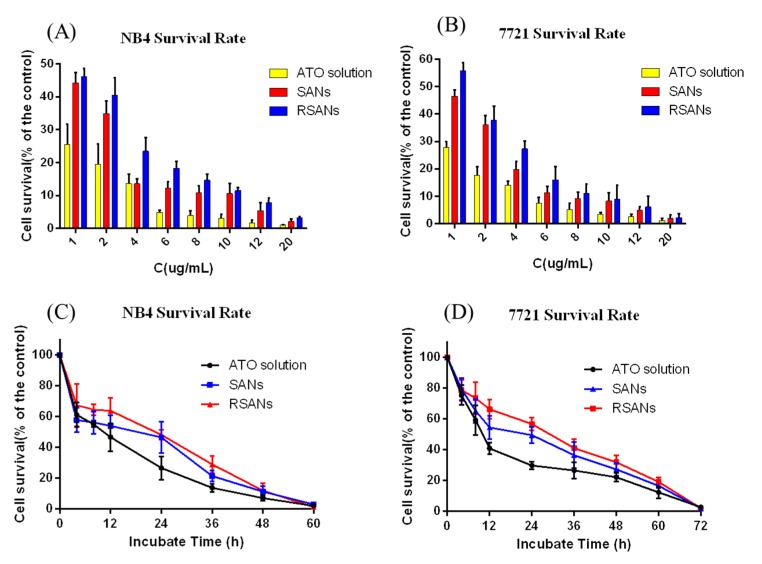
The inhibition effects on NB4 and 7721 cells after administration with different formulations. Cell survival of NB4 (**A**) and 7721 (**B**) cells after administration with different concentrations of ATO, SANs and RSANs for 24 h. Cell survival of NB4 (**C**) and 7721 (**D**) cells after administration with multiple groups at the ATO concentration of 1 μg/mL for 72 h. Data are shown as ± SD of the mean (*n* = 3).

Figure 9A,B showed that the inhibitory effects of free ATO, SANs, and RSANs on NB4 cells and 7721cells were all dose-dependent. With the increase of ATO concentration, the inhibition was significantly enhanced after 24 h. From Figure 9C,D, potent inhibition was observed at a lower concentration of 1 μg/mL. NB4 cells and 7721 cells were almost completely inhibited at 60 h and 72 h, respectively. At the same time point, the inhibitory intensity of the free ATO group, SANs group, and RSANs group decreased. However, because of the closed system of culture plate and sustained release nanoparticles, drugs in SANs and RSANs could be gradually released over time and eventually reaching the same effect as the free group. According to the results, it can be speculated that RSANs can avoid rapid elimination by the immune system in vivo with the inhibitory effect maintained.

### 3.9. In Vivo Toxicity and Safety Test

**Figure 10:**
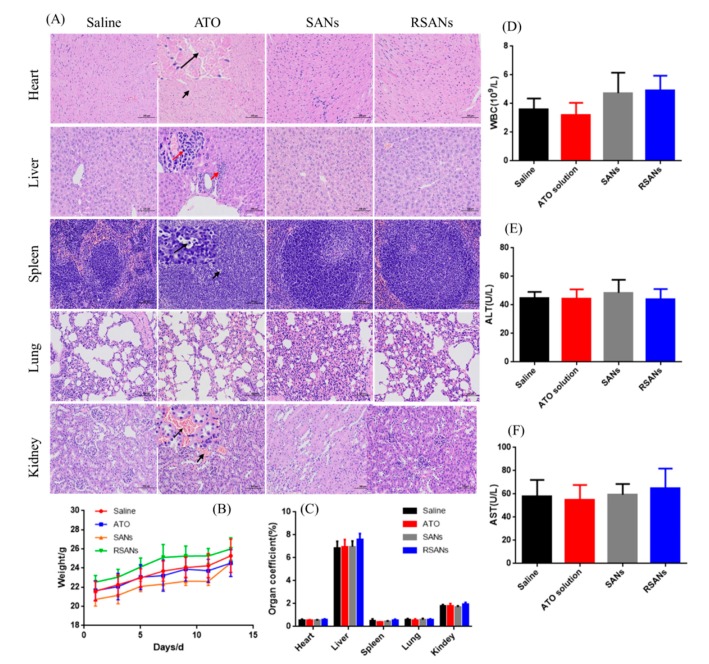
In vivo toxicity and safety evaluation. (**A**) H & E staining of primary organs under 100 μm. (**B**) Weight changes of nude mice during the experiment. (**C**) Organ coefficients, (**D**) Number of white blood cells, (**E**) ALT, and (**F**) AST of each group at the end of the experiment. Data are shown as ± SD of the mean (*n* = 5).

In acute toxicity test, some mice in the high concentration group died, and the surviving mice were inferior to other groups in terms of mental vitality, drinking, and feeding. During continuous administration, the average body weight of the mice in each group showed an increasing trend with no abnormal change, as shown in Figure 10B,C. It indicated that all the agents have no significant systemic toxicity. All hematological analysis results (Figure 10D–F) were within the normal range except for a slight decrease in the number of WBC in the free ATO group. Analysis of the main organ tissue sections after H & E staining showed that the SANs group and RSANs group were similar to the saline group, while the ATO group developed certain lesions (Figure 10A). The specific manifestation including a large number of cardiomyocytes was cytoplasmic loosely stained. Inflammatory infiltration was observed around the local portal area of the liver. The white pulp of spleen part conglutinated to each other with irregular shape and a small number of apoptotic bodies were seen. Moreover, local interstitial congestion could be observed in the kidney. According to the above results, it can be speculated that free ATO group can cause chronic toxicity if administered continuously in this concentration as compared to SANs group and RSANs group. The transient blood concentration of the nano-groups was reduced due to the sustained release, with a lower in vivo toxicity.

### 3.10. In Vivo Anti-Tumor Study

**Figure 11:**
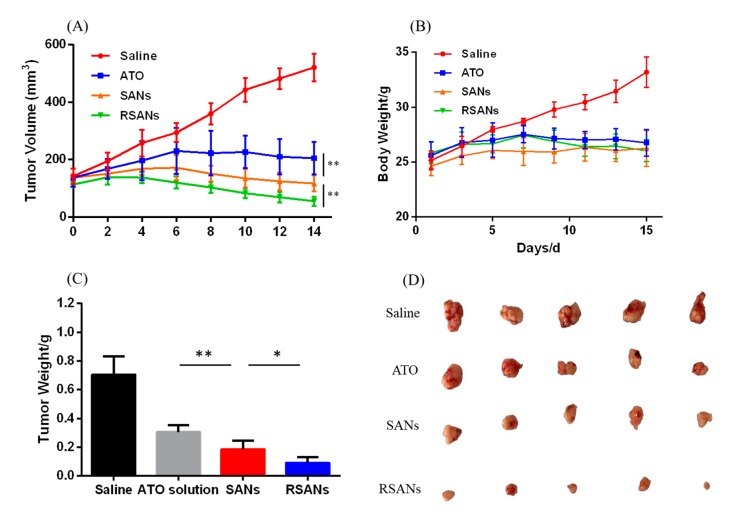
In vivo anti-tumor efficacy of formulation in the 7721 tumor bearing nude mice study. Changes of (**A**) tumor volume and (**B**) body weight during the period of treatment. (**C**) Average tumor weight after treatment. (**D**) Photograph of tumors collected after treatment. Data are shown as ± SD of the mean (*n* = 5). ** correspond to *p* < 0.01, * correspond to *p* < 0.05.

7221 tumor bearing nude mice were used to determine the anti-tumor efficacy of formulations. Figure 11 shows the results of different anti-tumor studies. Figure 11A showed the change in tumor volume during administration. In the saline group, the volume was gradually increased while the change trends of ATO group, SANs group, and RSANs group were first increased and then decreased (*p* < 0.01). The tumor mass and size at the end of the study were monitored as Figure 11C,D. According to the results, it could be speculated that the free ATO was quickly cleared in the body, the amount of drug reaching the tumor site was less than that of the other two groups, and that caused the lowest tumor inhibition efficacy. Over the whole treatment period, SANs group and RSANs group can achieve better tumor inhibition effects with the drug gradually released from the nanoparticles. At the same time, because of the wrapping of RBCM, RSANs were less likely to be cleared by the immune system than SANs, allowing more drugs to reach the tumor site. Figure 11B showed changes of body weight. By the end of the study, body weight of the saline group increased significantly, while the other three groups showed little variation, which was consistent with the results of the in vivo toxicity and safety test. It was further supported that the safety and low toxicity of SANs and RSANs, and the DDS can still improve efficacy of ATO.

## 4. Conclusions

The objective of this project was to realize sustained release of ATO and ensure the safety and efficacy of the DDS. SANs were prepared by coating ATO with SA, and then RSANs with a shell-core bilayer structure was obtained by wrapping the RBCM on the surface of the SANs. The nanoparticles were homogeneous with spherical structures and stable for 15 days with an average EE and DL of 14.31% and 4.98%, respectively. Compared to free ATO, the SANs and RSANs showed excellent sustained release in vitro for 3 days. Generally, biological characteristics of RBCM could be used to avoid the recognition of macrophages. Even when ingested by NB4 cells and 7721 cells, RSANs still could maintain its nuclear-shell structure. At the same time, in vitro safety test showed the safety of carrier materials and without observable toxicity to 293 cells or hemolysis and aggregation of red blood cells. Free drug can kill nearly half of cells at a low concentration, while RSANs can significantly reduce the toxicity of ATO. After continuous administration of ATO formulations through IV tail injection, the results revealed that the SANs and RSANs had no significant systemic, blood, and organ toxicity. Moreover, the RSANs could maintain inhibition of NB4 cells and 7721 cells. After in vivo administration, RSANs can avoid rapid clearance and exert their sustained release properties, thereby enough drugs can be transported to the treatment site and achieve the effect. This may allow RSANs greater advantages in the treatment of tumors. Therefore, it could be concluded that the Nano-drug delivery system can decrease the toxicity of ATO with high safety and the potential for treating APL as well as anti-hepatocarcinoma. Meanwhile, RSANs can prolong the duration of ATO in vivo, thus reducing administration times and enhancing patient compliance. In consequence, the DDS can be developed into a safe and sustained release delivery system for ATO.

## Figures and Tables

**Table 1:** Hemolysis test of RSANs.

Tube No.	1	2	3	4	5	6	7	8	9	10	11	12
2% RBC Suspension (mL)	2.5	2.5	2.5	2.5	2.5	2.5	2.5	2.5	2.5	2.5	2.5	2.5
Saline (mL)	0	2.5	2.4	2.3	2.2	2.1	2.0	1.9	1.8	1.7	1.6	1.5
Ultrapure Water (mL)	2.5	0	0	0	0	0	0	0	0	0	0	0
Samples (mL)	0	0	0.1	0.2	0.3	0.4	0.5	0.6	0.7	0.8	0.9	1.0

## Supplementary Materials

The following are available online at https://www.mdpi.com/1999-4923/12/1/21/s1, Table S1: Investigation of encapsulation efficiency and drug loading capacity.
