# Supplementary material for: Erythrocyte Membrane-Coated Arsenic Trioxide-Loaded Sodium Alginate Nanoparticles for Tumor Therapy
Source: Pharmaceutics. 2019 Dec 24;12(1):21. doi: 10.3390/pharmaceutics12010021 (PMC7022614; doi:10.3390/pharmaceutics12010021)
Supplement: Supplementary file 1 [file pharmaceutics-12-00021-s001.pdf]

pharmaceutics

Supplementary Information: Erythrocyte Membrane Coated Arsenic Trioxide Loaded-Sodium Alginate Nanoparticles for Tumor Therapy

Yumei Lian, Xuerui Wang, Pengcheng Guo, Yichen Li, Faisal Raza, Jing Su and Mingfeng Qiu

Table S1. Investigation of encapsulation efficiency and drug loading capacity.

Concentration of ATO (mg/mL)	Content of Arsenic (ppm)	SD (%)	RSD (%)	Encapsulation Efficiency (%)	Loading Capacity (%)
4	0.0391	0.0001	0.1546	4.82	1.01
6	0.0533	0.0001	0.2353	13.50	3.86
8	0.0704	0.0003	0.4884	14.31	4.98

With the increase of ATO concentration, both the encapsulation efficiency and drug loading capacity also increased. When ATO concentration was 10 mg/mL, the drug would precipitate out after dissolution, therefore ATO concentration was chosen as 8 mg/mL, the encapsulation rate was 14.31% and the drug loading capacity was 4.98%.
